# Do Affective Variables Make a Difference in Consumers Behavior Toward Mobile Advertising?

**DOI:** 10.3389/fpsyg.2016.02018

**Published:** 2017-01-03

**Authors:** María Pilar Martínez-Ruiz, Alicia Izquierdo-Yusta, Cristina Olarte-Pascual, Eva Reinares-Lara

**Affiliations:** ^1^Department of Business Administration, University of Castilla-La ManchaAlbacete, Spain; ^2^Department of Business Administration, University of BurgosBurgos, Spain; ^3^Department of Economics and Business Studies, Universidad de La RiojaLogroño, Spain; ^4^Department of Business Administration, Universidad Rey Juan CarlosMadrid, Spain

**Keywords:** antecedents, consequences, attitude, mobile advertising, permission marketing, emotions, feelings

## Abstract

Research into permission-based mobile marketing is increasingly common due to the widespread adoption of mobile technology and its use as a communication channel. Yet few studies have attempted to analyze the factors that determine attitudes toward mobile advertising while simultaneously considering: the links among them and consumers' intentions, behavior, and/or cognitive and affective variables simultaneously. The present research therefore sought to deepen understanding of the antecedents and consequences of attitudes toward permission-based mobile advertising. More specifically, it sought to identify the antecedents of attitudes toward mobile advertising and the bridges between these attitudes and consumers' intentions upon receiving advertising on their mobile devices. To this end, a causal model was proposed and tested with a sample of 612 mobile phone users that was collected from a panel of Spanish adults who receive advertising on their mobile phones in the form of SMS text messages. The structural model used was validated using the partial least squares (PLS) regression technique. The results show that the greatest influence was that exerted by positive emotions on feelings, suggesting that positive emotions have an indirect effect on attitude toward mobile advertising. This influence was even greater than their direct effect. Another important, though less powerful, effect was the influence of attitude on behavioral intentions to receive mobile advertising. In contrast, the influence of cognitive variables on attitude was less relevant.

## Introduction

The high potential that mobile devices offer as a medium for delivering advertising to consumers is based on several key factors. First, companies tend to believe that mobile advertising has a high capacity to reach almost anyone anywhere at any time (Haghirian and Madlberger, 2005; Richard and Meuli, 2013). This belief is supported, among other things, by the high penetration rate of mobile phones among end users, which became quite significant in the 1990s (e.g., Khalifa and Shen, 2008; Zhang and Mao, 2008). Today, it continues to increase worldwide, making it possible to understand the large amounts of time consumers spend on their devices (e.g., Bart et al., 2014).

The use of this type of mobile technology enables relatively more personal and interactive communication with consumers (e.g., Bauer et al., 2005; Sanz-Blas et al., 2015), as well as the development of specific differentiated strategies to target them (Drossos et al., 2007; Sultan et al., 2009; Olarte-Pascual et al., 2014). Moreover, due to the proliferation and growth of localization technologies, mobile advertising makes it possible to send messages to subscribers based on their geographic location. Thus, mobile advertising can range from completely undifferentiated (mass) messages to, where desired, messages tailored to each individual (Richard and Meuli, 2013). This technological resource opens the door to new business opportunities in the field of mobile advertising, offering considerable competitive advantages in terms of customization and of presenting the most relevant information to each consumer (Bauer et al., 2005; Kim and Han, 2014). However, it has also contributed to growing consumer concerns about issues related to the protection of privacy and personal data (Haghirian and Madlberger, 2005). These concerns illustrate the need for permission-based mobile advertising (PBMA), that is, advertising requiring individuals to give their permission before they can receive any type of advertising message (Godin, 1999; Varnali and Toker, 2010).

Indeed, numerous studies have confirmed this fact. For instance, Barwise and Strong (2002) observed that requesting permission in advance influenced the effectiveness of SMS text messaging as an advertising medium for reaching young adults. Likewise, following a comprehensive review of the relevant literature on mobile marketing, Varnali and Toker (2010) found that, among the top six best practices in mobile marketing, requesting permission, and addressing consumers' security and privacy concerns are particularly important. Baek and Morimoto (2012) corroborated this importance, noting that consumers' perception of the behavioral intentions behind text message advertising was influenced by the concern shown for whether their privacy was preserved. In light of all these findings, it is unsurprising that there is some consensus among mobile advertising industry operators that one of the keys to the success of a mobile marketing campaign is that it not be intrusive (Soroa-Koury and Yang, 2010).

In order to provide a solid theoretical basis for examining the adoption of mobile advertising, this paper draws on two schools of thought regarding the nomological structure (Lee, 2009) of the Theory of Reasoned Action (TRA) (Fishbein and Ajzen, 1975): (i) the Technology Acceptance Model (TAM) (Davis, 1989) and (ii) the Theory of Planned Behavior (TPB) (Ajzen, 1991). Since TAM and TPB have been used in many studies to predict and understand user perceptions of systems use and the probability of adopting an online system (Gefen et al., 2003; Wu and Chen, 2005; Hsu et al., 2006), they are the most appropriate tools for understanding mobile advertising adoption. This investigation, similar to others, (Mathieson, 1991; Igbaria et al., 1995; Taylor and Todd, 1995; Lee, 2009), proposes to integrate both models, TAM, and TPB, in order to provide a more comprehensive model of mobile advertising.

The TAM is an adaptation of the TRA by Fishbein and Ajzen (1975) and was developed by Davis (1989) to explain acceptance of information technology for different tasks. This model hypothesizes that system use is directly determined by behavioral intention of use, which is in turn influenced by users' attitudes toward using the system and the perceived usefulness of the system. Attitudes and perceived usefulness are also affected by perceived ease of use. A critical review of TAM has revealed that there is a need to include other components in order to provide a broader view and a better explanation of Information Technology (IT) adoption. As a matter of this fact, since TAM originated in work contexts where emphasis was mainly placed on variables related to job performance, it seems reasonable to give consideration to affective variables that might contribute to acceptance of technologies in more hedonic scenarios, such as the context in which consumers use their mobile devices (Van Der Heijden, 2004; Abad et al., 2010). Moreover, factors related to human and social change processes should be also incorporated. For example, in Information System (IS) literature, the TAM (Davis, 1989), the extended Technology Acceptance Model (TAM2) (Venkatesh and Davis, 2000) and the Unified Theory of Acceptance and Use of Technology (UTAUT) (Venkatesh et al., 2003) are used to explain possible adoption and acceptance patterns of new technologies among consumers. In all these models, concepts like relative advantage, compatibility, complexity, and observability, as well as perceived risk, perceived usefulness, subjective norm, and perceived ease of use play a key role in these approaches.

In the same way, in the context of mobile communications, few studies have taken a holistic approach of the interrelated antecedent factors. Instead, most research has sought to assess only one cognitive dimension. From a joint cognitive-affective perspective, the theoretical background underlying the assumptions of the model for attitudes toward mobile advertising includes eminently cognitive variables (Karjaluoto et al., 2008; Soroa-Koury and Yang, 2010).

In light of these limitations identified in the literature, the present research aimed to fill this gap by delving deeper into the study of the advisability of treating attitudes toward mobile advertising as a two-dimensional variable including both cognitive and affective factors, and the antecedents and consequences of attitude toward PBMA. More specifically, we have proposed a model to meet the stated aims and to analyze the antecedents of attitudes toward mobile advertising and the relationship between attitude and intention in consumers who receive advertising via their mobile devices.

With these ideas in mind, the remainder of the paper is organized as follows. First, it reviews the relevant literature underlying each of the proposed research hypotheses. Next, it describes the proposed research methodology based on the study of a representative sample in Spain. Finally, it reports the main results and conclusions of the research, placing special emphasis on the implications for businesses and taking into account certain limitations.

## Conceptual framework

This section will review the relevant literature with a view to selecting the most important antecedents and consequences of attitude toward PBMA. To this end, amongst the antecedents of attitude toward mobile advertising, it will distinguish between cognitive and affective variables and between utilitarian and hedonic ones. This approach is in keeping with Yang et al. (2013), who proposed that taking these classifications into account facilitates a deeper understanding of the possible effects of mobile advertising. It moreover addresses the criticism TAM has received for its excessive dependence on external factors (e.g., perceived usefulness) and exclusion of internal ones (e.g., emotions). Such criticism would seem to indicate that TAM alone is not enough to explain consumers' responses to mobile advertising, but rather affective variables likely to influence attitude formation must be considered too. This is especially true given that TAM originated in work contexts, in which emphasis is placed on variables related to job performance. In contrast, in more hedonic scenarios, such as the context in which consumers use their mobile devices, consideration must also be given to the affective variables that might contribute to acceptance of the technology in question (Van Der Heijden, 2004; Abad et al., 2010). Moreover, while the benefits of considering both cognitive and affective factors in order to better understand peoples' appraisals have been widely recognized in the literature (Van Waterschoot et al., 2008; Levav and McGraw, 2009; Zielke, 2011), it is not yet known how these factors combine to influence attitudes toward mobile advertising and, thus, the intention to receive it. In this context, the first aim of this paper is to fill these gaps. As noted, both cognitive and affective factors influence subjects' appraisals (Dean et al., 2008). Affect and cognition take place through an interlocked dual system that comes together in natural human behavior (Boehner et al., 2007). Moreover, some authors (e.g., Vincent and Harper, 2003; Vincent and Haddon, 2004) have found that, even in some work settings, employees use their mobile phones for their social relationships with partners, family and friends more than with clients.

Given these ideas, it should be noted that the assumptions on which this paper is based are also consistent with the relevant literature in the field of social psychology, which establishes that attitude is influenced by both cognitive and affective variables (e.g., Bagozzi and Burnkant, 1985; Chaiken and Strangor, 1987; Weiss and Cropanzano, 1996).

### Antecedents of attitude toward mobile advertising

Perceived usefulness is generally understood to refer to the judgment customers make regarding a product's utility based on their perceptions of what they give and what they receive (Zeithaml, 1998). It thus consists of a perceived preference for and evaluation of the product's attributes, attribute performances, and the consequences arising from its use that allow the consumer to achieve his or her goals in different use situations (Woodruff, 1997).

The importance of this concept is evident in the numerous studies conducted in recent years (e.g., Wu and Wang, 2005; Kim et al., 2008; Flavián et al., 2009; Hess et al., 2014; Izquierdo-Yusta et al., 2015; Olarte-Pascual et al., 2016). By way of example, attention should be drawn to the perceived value model proposed by Zeithaml (1998), which served as the inspiration for several subsequent studies highlighting the influence of perceived value on consumers' behavioral intentions (Dodds et al., 1991; Grewal et al., 1998; Sweeney and Soutar, 2001). Among these studies, attention should be called to those carried out based on TAM. TAM clearly assigns fundamental importance to perceived usefulness, proposing it as a key antecedent of attitude toward the use of a given technology.^1^ From this perspective, perceived usefulness becomes a very important variable for understanding user behavior in relation to mobile advertising. For instance, in their study using the extended TAM model (TAM2),^2^ Venkatesh and Davis (2000) found that perceived usefulness predicted attitude toward mobile advertising, compared to other variables that barely influenced it at all. Kavassalis et al. (2003) observed that when consumers perceived a benefit in receiving advertising messages on their mobile phones, they were more willing to accept such advertising.

In a study analyzing the acceptance of SMS advertising among young people between the ages of 21 and 35, Zhang and Mao (2008) found that perceived usefulness was one of the most important variables for predicting the intention to use that advertising. In the context of permission-based mobile marketing, Karjaluoto et al. (2008) found that the perceived usefulness of mobile communications explained attitude toward mobile advertising. Likewise, in their analysis of a sample of 343 university students, Soroa-Koury and Yang (2010) found that perceived usefulness predicted attitude toward mobile advertising.

In light of the influence that these studies have shown perceived usefulness to have on attitude, the following research hypothesis was proposed:

H1. Perceived usefulness positively and significantly influences attitude toward mobile advertising.

The subjective norm, or influence of reference groups on an individual, is often used as a variable to address the importance of social context with regard to behavior. Its influence on attitudes and behavioral intentions has been widely demonstrated in the academic literature (c.f., Fishbein and Ajzen, 1975; Bearden and Etzel, 1982; Bagozzi, 2000; Pelegrín-Borondo et al., 2016; Versluis and Papies, 2016).

In order to measure the influence of this variable, Fishbein and Ajzen (1975) identified the potential use of two variables: normative belief, referring to what other people want the individual to do, on the one hand, and the motivation to comply with different reference groups on the other. To this end, Peter and Olson (2005) highlighted the influence of two main reference groups: normative ones, such as parents and peers (Fitzgerald and Arndt, 2002), and comparative ones, such as idols (Childers and Rao, 1992).

TAM2 highlights the influence of the subjective norm on the intention to adopt a new technology. From this perspective, Venkatesh and Davis (2000) observed that the subjective norm has a stronger direct impact on usage intentions than other variables, such as perceived usefulness. However, drawing on previous studies conducted using TAM2 (e.g., Venkatesh and Davis, 2000), Soroa-Koury and Yang (2010) suggested that social norms are likely to influence the perceived usefulness of mobile advertising. Subsequent studies (e.g., Bauer et al., 2005; Muk and Babin, 2006; Rohm and Sultan, 2006; Lee et al., 2013) have confirmed the positive influence of reference groups on the intention to participate in mobile marketing.

However, few papers have focused on the influence of the subjective norm on attitude toward mobile advertising, considering this attitude to be an antecedent of intention and behavior. In light of the ideas discussed above, the following research hypothesis was proposed, referring to the positive and significant influence of social norms on attitude toward mobile advertising:

H2. The subjective norm positively and significantly influences attitude toward mobile advertising.

In the 1980s, individuals' emotions began to be associated with their decision-making processes and ceased to be considered external elements likely to hinder the optimal functioning of the process (Zajonc, 1980). Moreover, the literature has demonstrated the ability of emotions to stimulate individuals' behavioral intentions (Bagozzi et al., 1999; Zajonc, 2000) and the existence of groups of people who tend to react similarly to certain emotions (Mano, 2004; Pelegrín-Borondo et al., 2016). Thus, in general, objects that cause pleasant sensations are evaluated positively, while those that cause unpleasant sensations are evaluated negatively (Pham, 2007; Bargh, 2013). Likewise, individuals tend to avoid unpleasant situations, engage in activities they find pleasant (Bagozzi et al., 1999; Mano, 2004), and select alternatives that make it easier for them to experience positive, rather than negative, emotions (Bower and Cohen, 2014).

With regard to purchase decision-making processes, Oliver (2014) has shown that when consumers assess the available alternatives and find that the service quality exceeds their expectations, it influences their emotions and pleasure, generating a feeling of delight with the service and positively impacting their intention to repeat their choice. White and Yu (2005) found that positive emotions were positively correlated with the tendency to speak favorably about products, while negative ones encouraged unfavorable communication and the search for available alternatives. Likewise, Forgas and Ciarrochi (2001) found that positive moods enhanced peoples' assessments of products, while negative ones lowered them. Similarly, O'Neill and Lambert (2001) showed that surprise and enjoyment positively affect product evaluations and the act of choosing. Therefore, it can generally be stated that consumers have a natural tendency to make choices that minimize the likelihood of experiencing negative emotions (e.g., Elliott, 1998; Schwarz, 2000; Carstensen and Mikels, 2005).

In the field of online advertising, WAM assumes the existence of three antecedents—entertainment, irritation, and informativeness—as the main determinants of attitudes toward online advertising (Ducoffe, 1996). In this line of research, Tsang et al. (2004) observed that consumers prefer entertaining content over other types of content (such as informative content) when it comes to accepting mobile services. In general, the content and form of advertisements are important predictors of their value, and they are critical to the effectiveness of online advertising (e.g., Ducoffe, 1996; Berger and Milkman, 2012; Teixeira et al., 2012). These findings are consistent with those of other studies carried out decades ago in the sphere of conventional advertising, such as Mitchell and Olson (1981) and Shimp (1981), who observed that interesting and enjoyable advertisements had a positive impact on the brand, or Schlosser et al. (1999), who reported that attitudes toward online advertising were influenced by enjoyment, informativeness, and utility.

However, some studies have shown that certain emotions, such as irritation, negatively impact advertising avoidance by consumers (Ducoffe, 1996; Martí Parreño et al., 2013; Yang et al., 2013). Elliott and Speck (1998) observed that individuals reported negative responses (whether ad avoidance or the development of negative feelings toward the ad) when they were shown cluttered advertisements or when the ads hindered their search for information. More recently, Ünal et al. (2011) corroborated the fact that, if a mobile advertisement is sent with permission, and it is entertaining, informative, reliable, and personalized, it positively affects the creation of positive attitudes toward advertising, although they found some differences in the relationships between attitude, intention, and behavior depending on whether the recipients of the advertising were youths or adults.

Specifically in the field of mobile communication, rather than highlighting the functionality of the communication that mobile devices enable, many studies have stressed the emotional attachment that can potentially be established between the user and his or her mobile device (e.g., Vincent, 2005). From this perspective, Kolsaker and Drakatos (2009) examined the relationship between the strength of the emotional attachment to the mobile device, the perceived benefits of mobile advertisements, and receptiveness toward them, confirming that users with a strong emotional attachment to their mobile devices are more receptive and perceive greater potential benefits in mobile advertising than the rest.

Likewise, emotions are closely related to other affective states, such as feelings. Feelings are the conscious assessment of the perceived body state during an emotional response. In other words, feelings are the results of emotions. Therefore, feelings occur when the brain is aware of the bodily change occurring as a result of a given emotion and are thus subsequent to emotions. Moreover, although emotions are more intense, they are also more short-lived; consequently, the optimal strategy should focus not so much on generating an emotional attachment as on achieving a sentimental one, which is more enduring (Bechara et al., 2000).

Based on the above, the following working hypotheses were proposed:

H3. Positive emotions positively and significantly influence attitude toward mobile advertising.
H4. Positive emotions positively and significantly influence feelings toward mobile advertising.
H5. Negative emotions negatively and significantly influence attitude toward mobile advertising.
H6. Negative emotions negatively and significantly influence feelings toward mobile advertising.
H7. Feelings positively and significantly influence attitude toward mobile advertising.

### Consequences of attitude toward mobile advertising

Attitude toward advertising has been extensively studied in recent decades in academia (e.g., Shavitt et al., 1998; Dutta-Bergman, 2006), where it is usually considered to be an antecedent of individuals' behavior and final decisions from the perspective of the aforementioned theories. The empirical evidence obtained has underscored the suitability of using the variable attitude as a determinant of the intentions and behavior of mobile phone users when it comes to accepting advertising. An initial study by Kim and Hunter (1993) on the links between attitude and behavioral intention showed that attitude is positively related to intention.

Subsequently, Lee et al. (2006) observed that favorable attitudes toward mobile advertising, correlated with strong motives, significantly influence intentions and positive actions. Barutçu (2007) found that users have positive attitudes toward certain mobile marketing tools, including advertising. In the context of TAM, Karjaluoto et al. (2008) found that attitude explained a considerable amount of the intention to receive advertising messages from a company, and that this relationship was stronger in women. Based on TAM2, Soroa-Koury and Yang (2010) and Xu (2006) found that attitude toward mobile advertising significantly predicted the intention to adopt mobile advertising. Although most of the studies conducted in this line of research have observed this positive relationship, in some cases, it could not be verified.

Based on these prior studies, in which attitude was generally found to positively influence intention, the following research hypothesis was proposed:

H8. The more positive the attitude toward mobile advertising is, the greater the intention to receive mobile advertising.

**Figure 1:**
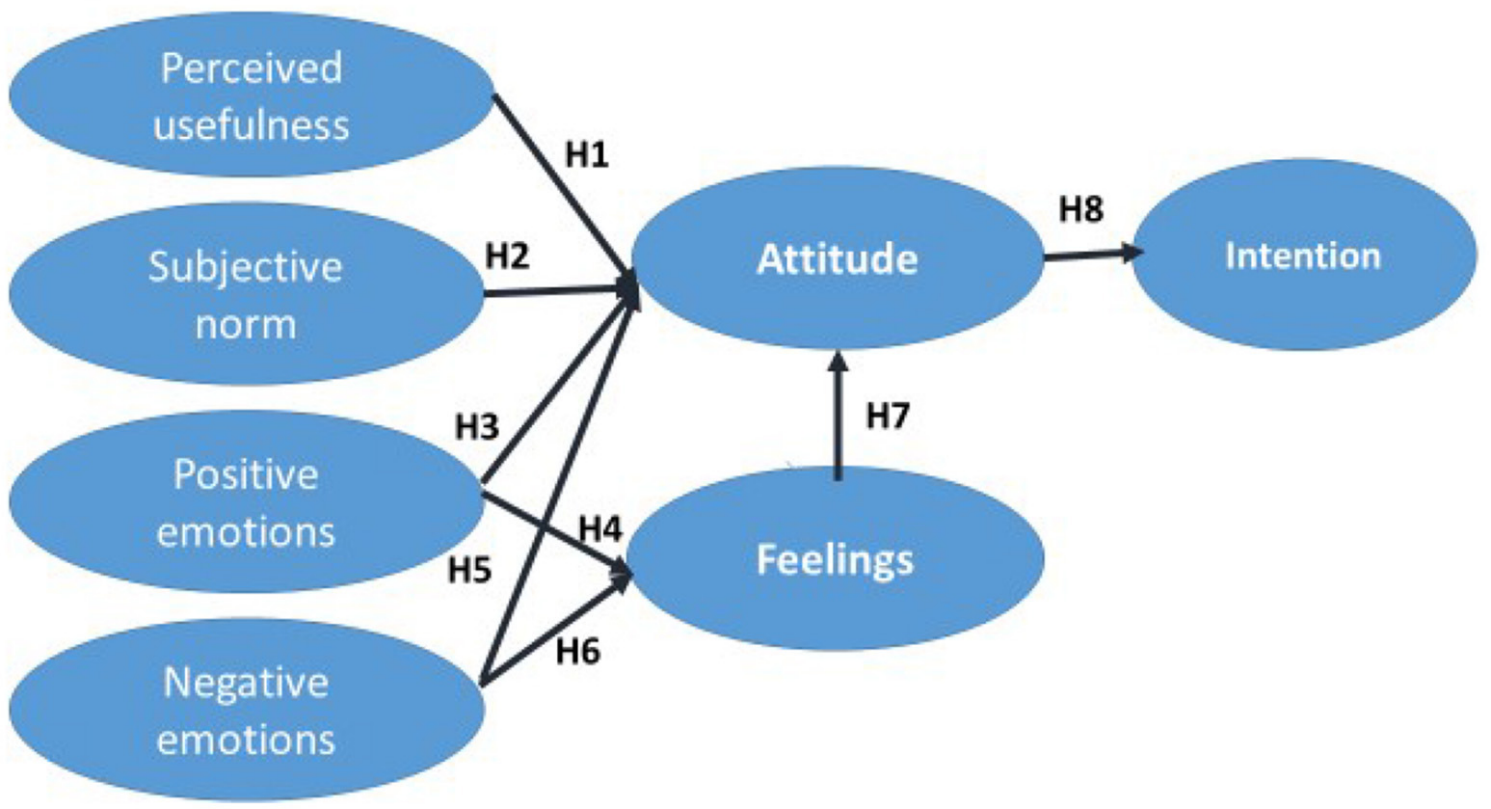
**Graphical representation of the proposed conceptual model**.

Based on the proposed hypotheses, a theoretical model was defined that integrates the various variables influencing attitudes and intentions with regard to mobile advertising in text format (Figure 1).

## Research methodology

To carry out the proposed empirical research, a sample was collected from a panel of Spanish adults who receive advertising on their mobile phones in the form of SMS text messages. This was achieved with the technical support of Cint Panel Exchange, as it both enables access to a broad sample of consumers representative of the Spanish market and ensures a high level of quality. The data was obtained in two steps: (1) randomized 9,000 sending invitations with a response rate of 26% and (2) select only individuals receiving mobile advertising by taking into account the structure of the Spanish population by gender and age. The sample was of 612 individuals who were representative of the Spanish adult population and received advertising on their mobile phones. 92.3% of the sample had been using a mobile phone for more than 5 years, and 35.9% had more than one mobile. The most common mobile phone brand was Nokia (40.5%), followed by Samsung (18.8%). The predominant sociodemographic characteristics were: 51.8% were women; 33.8% were between the ages of 35 and 44; 36.6% had a college education; 60.3% were married; and maximum income level was 1201–1800 euros per month. Table 1 shows the technical details of the research.

**Table 1:** **Technical details of the research**.

**Universe**	**Individual mobile-phone users and recipients of SMS advertising**
Sampling procedure	Random
Type of questionnaire	Self-administered survey based on a structured questionnaire administered online (Cint Panel Exchange)
Scope	Spain
Actual sample	612
Sampling error	±4.04%
Confidence level	95% (*Z* = 1.96)
Maximum possible variance	*P* = *q* = 50%
Fieldwork	June 2011

Two consecutive rounds of pre-testing were conducted to verify proper comprehension of the questionnaire and the adaptation thereof to the aims of the research. Table 2 provides a description of the variables included in each construct. All variables were measured by means of a 5-point Likert scale, where 1 was strongly disagree and 5 was strongly agree.

**Table 2:** **Table 2 Measurement scales**.

**Factor**	**Variables/items**	**Scale**	**References questionnaire**
Perceived usefulness	PU1: Real-time delivery	1–5 Likert scale	Dodds et al., 1991; Woodruff, 1997; Grewal et al., 1998; Zeithaml, 1998; Sweeney and Soutar, 2001; Gallarza and Gil, 2006; Choi et al., 2008; Flavián et al., 2009
	PU2: Ability to access it whenever I want		
	PU3: Ability to access it wherever I want		
	PU4: Offers additional benefits		
	PU5: Other advantages		
Subjective norm	SN1: I use mobile advertising because my friends do		Fishbein and Ajzen, 1975; Bearden and Etzel, 1982; Davis, 1989; Ajzen, 1991; Childers and Rao, 1992; Bagozzi, 2000; Fitzgerald and Arndt, 2002; Bauer et al., 2005; Muk and Babin, 2006; Peter and Olson, 2005; Rohm and Sultan, 2006
	SN2: I tell my friends about the advertising sent to my phone		
	SN3: My reference group thinks I should receive advertising on my phone		
	SN4: I receive advertising on my phone because my friends do too		
Emotions (+)	EM(+)1: It is enjoyable		Tsang et al., 2004; Xu, 2006; Izquierdo-Yusta et al., 2015; Olarte-Pascual et al., 2016
	EM(+)2: It is interesting		
	EM(+)3: It is motivating		
	SENT2: It is useful		
	SENT3: It is convincing		
	SENT4: It is credible		
	SENT5: It is influential		
	SENT6: It is persuasive		
Attitude	ATT1: It catches my attention		Davis, 1989; Ajzen, 1991; Shavitt et al., 1998; Tsang et al., 2004; Dutta-Bergman, 2006; Lee et al., 2006; Xu, 2006; Soroa-Koury and Yang, 2010
	ATT2: I find it entertaining		
	ATT3: It influences my buying behavior		
	ATT4: I like it		
Intention	INT1 Intention to receive mobile advertising		Tsang et al., 2004; Choi et al., 2008

With regard to ethics approval: (1) all participants were given detailed written information about the study and procedure; (2) no data directly or indirectly related to the subjects' health were collected and, thus, the Declaration of Helsinki was not generally mentioned when the subjects were informed; (3) the anonymity of the collected data was ensured at all times; and (4) no permission was obtained from a board or committee—voluntary completion of the questionnaire was taken as consent for the data to be used in research.

## Results

The structural model used (Figure 1) was validated using the partial least squares (PLS) regression technique. PLS path modeling is frequently used in researches referred to TRA, TAM, TPB (Henseler et al., 2016). Although there is a debate about which technique is more appropriate, if PLS (e.g., Wold, 1982; Lohmöller, 1989), or covariance based structural equation modeling (SEM) (e.g., Jöreskog and Wold, 1982), we decided to use PLS due to the higher benefits it provides in this kind of researches (e.g., possibility to use with both reflective and formative constructs; small samples).

The model was estimated using the statistical software SmartPLS 2.0, and the significance of the parameters was established by means of bootstrap resampling. To ensure convergent validity, all indicators whose factor loading was not significant or was <7 were eliminated. Thus, the resulting model presented no reliability issues with regard to any of the established criteria (Cronbach's alpha, composite reliability, and average variance extracted) (see Table 3).

**Table 3:** **Reliability and convergent validity of the model**.

**Factor**	**Indicator**	**Loading**	***T*-value**	**Cronbach's α**	**Compound reliability**	**AVE**
Perceived usefulness	PU1: Real-time delivery	0.814^***^	40.56	0.9105	0.9319	0.7327
	PU2: Ability to access it whenever I want	0.851^***^	52.53			
	PU3: Ability to access it wherever I want	0.859^***^	60.80			
	PU4: Offers additional benefits	0.884^***^	103.33			
	PU5: Other advantages	0.878^***^	92.38			
Subjective norm	SN1: I use mobile advertising because my friends do	0.855^***^	50.97	0.8869	0.9219	0.7472
	SN2: I tell my friends about the advertising sent to my phone	0.801^***^	48.35			
	SN3: My reference group thinks I should receive advertising on my phone	0.912^***^	128.12			
	SN4: I receive advertising on my phone because my friends do too	0.885^***^	71.5670			
Emotions (+)	EM(+)1: It is enjoyable	0.921^***^	103.33	0.9054	0.9407	0.8409
	EM(+)2: It is interesting	0.924^***^	122.81			
	EM(+)3: It is motivating	0.905^***^	83.40			
Emotions (−)	EM(−)1: It is irritating	0.948^***^	61.68	0.6809	0.8489	0.7397
	EM(−)2: It is misleading	0.762^***^	16.90			
Feelings	SENT1: It is informative	0.785^***^	42.58	0.9077	0.9292	0.6878
	SENT2: It is useful	0.826^***^	46.46			
	SENT3: It is convincing	0.695^***^	20.47			
	SENT4: It is credible	0.877^***^	81.01			
	SENT5: It is influential	0.887^***^	78.65			
	SENT6: It is persuasive	0.889^***^	93.44			
Attitude	ATT1: It catches my attention	0.884^***^	81.96	0.8976	0.9288	0.7654
	ATT2: I find it entertaining	0.846^***^	47.63			
	ATT3: It influences my buying behavior	0.852^***^	49.52			
	ATT4: I like it	0.915^***^	116.78			

*** *p < 0.01*.

Discriminant validity was assessed using the average variance extracted for each factor, taking into account that it should be greater than the squared correlation between each factor pair (Fornell and Larcker, 1981), as shown in Table 4.

**Table 4:** **Discriminant validity**.

	**Attitude**	**Emotions (+)**	**Intention**	**Subjective norm**	**Feelings**	**Emotions (-)**	**Perceived Usefulness**
Attitude	**0.874**						
Emotions (+)	0.8383	**0.9170**					
Intention	0.7366	0.7231	**0.860**				
Subjective norm	0.7033	0.6236	0.4915	**0.880**			
Feelings	0.8427	0.9100	0.7403	0.5964	**0.829**		
Emotions (-)	−0.3986	−0.4236	−0.4152	−0.1548	−0.360	**0.860**	
Perceived usefulness	0.7356	0.6823	0.6050	0.5759	0.6999	−0.2958	**0.0855**

*The off-diagonal numbers are the estimated correlations between the factors. The on-diagonal numbers in bold are the square roots of the average variances extracted*.

Once the measuring tool's psychometric properties had been evaluated, PLS was used to estimate the structural model synthesizing the proposed hypotheses shown in Figure 1. The same criterion used to determine the significance of the parameters (612 bootstrap subsamples the same size as the original) was used.

To assess the structural model's predictive ability, the criterion proposed by Falk and Miller (1992) was used, whereby the *R*^2^ of each dependent construct must be >1. Lower values, even if significant, should not be accepted. It is thus possible to determine whether or not the proposed hypotheses are supported, considering the significance of the estimated standardized regression coefficients (see Table 5).

**Table 5:** **Testing of hypotheses**.

**Hypothesis**	**Standardized β**	**Bootstrap *t*-value**
H1: Perceived usefulness > Attitude	0.182^***^	6.297
H2: Subjective norm > Attitude	0.251^***^	9.4373
H3: Positive emotions > Attitude	0.195^***^	7.756
H4: Positive emotions > Feelings	0.924^***^	82.966
H5: Negative emotions > Attitude	−0.096^***^	4.779
H6: Negative emotions > Feelings	0.0031^N.S.^	1.495
H7: Feelings > Attitude	0.353^***^	3.834
H8: Attitude > Intention	0.737^***^	35.181

*R^2^ Feelings = 0.831; R^2^ Attitude = 0.810; R^2^ Intention = 0.543*;

*** *p < 0.01; NS = not significant*.

The results show that the most important effects were those generated by positive emotions on feelings (β = 0.924; *p* < 0.01; H4) and by attitudes toward mobile advertising on the intention to receive mobile advertising (β = 0.737; *p* < 0.01; H8). The positive and significant influences of feelings on attitude (β = 0.353; *p* < 0.01; H7), the subjective norm on attitude (β = 0.251; *p* < 0.01; H2), positive emotions on attitude (β = 0.195; *p* < 0.01; H3) and perceived usefulness on attitude (β = 0.182; *p* < 0.01; H1) were less important. Finally, negative emotions were found to exert a minor (negative) influence on attitude (β = −0.096; *p* < 0.01; H5). Therefore, all of the research hypotheses were supported, except H6, referring to the negative influence of negative emotions on feelings.

These results underscore the strong influence positive emotions can have on attitude toward mobile advertising, especially indirectly through feelings. This is a very positive finding for companies that engage in mobile advertising: since feelings last longer than emotions, it is very favorable that positive emotions help to strengthen feelings. Companies should thus try to foster sentimental ties by conveying positive emotions in their mobile advertising messages, as that was found to be the strongest relationship established among the variables. The influence of attitude on the intention to receive mobile advertising was likewise considerable. This finding sheds light on how attitude largely translates to the intention to accept mobile advertising.

The subjective norm and perceived usefulness had less of an influence on attitude. Therefore, the most important variables were those related to affective aspects of the consumers. This finding helps to confirm the aforementioned criticism of TAM, which emphasizes the need to take more affective aspects of consumers into account. Not in vain, to understand these results, it is important to consider the hedonic context in which the present research was carried out.

## Conclusions

This paper has offered a joint analysis of the antecedents and consequences of attitude toward mobile advertising, based on a context of permission-based mobile marketing, and taking into account the precepts of models such as TAM or WAM. In so doing, it sought to fill an identified gap in the literature with regard to the joint study of these factors.

To this end, the relevant literature was reviewed, in order to identify the most important variables from both a cognitive and affective perspective. Among the cognitive variables conventionally considered to be antecedents of attitude under TAM (or, where applicable, TAM2), perceived usefulness and the subjective norm stood out; among the affective variables, special attention should be called to emotions—both positive and negative—and feelings. Finally, the most important consequence of attitude toward mobile advertising was the intention to receive it. A conceptual model was proposed and tested with a sample of 612 mobile phone users and recipients of text-message advertising.

The findings made it possible to measure attitudes toward mobile advertising, as well as their antecedents and consequences. Specifically, the greatest influence in the model was found to be that exerted by positive emotions on feelings, which refers to the indirect influence that positive emotions can have on attitude toward mobile advertising. This influence was found to be much greater than that exerted by positive emotions directly. Somewhat smaller, but nevertheless very important, was the influence of attitude on the behavioral intention to receive mobile advertising.

Additionally, the influence of cognitive variables on attitude was found to be far less important. In order to understand these results, several factors must be taken into account. First and foremost, as several authors have shown, TAM was originally applied in work contexts; however, the contexts in which end users most often use their mobile devices tend to be hedonic.

These findings clearly point to interesting opportunities for companies that engage in mobile advertising, especially since they underscore the importance of the role of affective variables, such as emotions and feelings, in consumer behavior. They are moreover perfectly consistent with the precepts of the latest approaches and theories in marketing, such as marketing 3.0, which suggests that the discipline of marketing should focus on meeting the full range of individual needs (cognitive, affective, spiritual, etc.). Furthermore, this paper has shown that positive emotions have a much stronger effect than negative ones. Although the latter should be taken into account insofar as they might indirectly influence the intention to receive mobile advertising, companies should focus their resources on emphasizing positive emotions and feelings toward mobile advertising rather than on mitigating the consequences of negative ones. They should moreover consider the strong influence of attitude on the intention to receive mobile advertising.

Therefore, since positive emotions were found to have a greater impact on attitude toward mobile advertising than any other variable, it can be concluded that, in mobile advertising, consumers' affective variables really make a difference. In this regard, the importance of mobile as a medium not only for conveying specific value propositions and offers, but also for carrying out other types of communication actions is clear. For instance, companies may find that mobile can be a very valuable medium when it comes to trying to build and even consolidate a certain brand image. In particular, they might try to achieve this by forging strong emotional bonds with consumers, which can then be transformed into feelings in the long term.

Finally, these findings are consistent with the field of social psychology, which holds that both cognitive and affective variables can influence attitude (e.g., Bagozzi and Burnkant, 1985; Chaiken and Strangor, 1987; Weiss and Cropanzano, 1996). More specifically, they are consistent with the influence of certain positive emotions on attitude toward mobile advertising reported elsewhere (e.g., Tsang et al., 2004; Ünal et al., 2011; Ruiz-Mafé et al., 2014). Tsang et al. (2004) also showed that the perceived irritation, along with the perceived entertainment, information, and credibility of mobile advertising, influenced attitude toward it.

With regard to limitations pointing to new avenues of research, this paper only considered recipients of text-message mobile advertising. It would be interesting to replicate this study considering a larger number of formats and mobile advertising media due to the spread of 3G and 4G technologies (such as advertising on web pages optimized for mobile devices, mobile apps, etc.) and the development of wearables (watches, glasses, etc.). Finally, future research could aim to identify those sociodemographic variables of the target audience that help to explain differences between groups. For instance, does age affect the influence of the subjective norm? Or does the influence of emotions depend on gender?

## Author contributions

All authors listed, have made substantial, direct and intellectual contribution to the work, and approved it for publication.

### Conflict of interest statement

The authors declare that the research was conducted in the absence of any commercial or financial relationships that could be construed as a potential conflict of interest.

## Footnotes

^1^In addition to perceived usefulness, TAM tends to include the variable *perceived ease of use*. However, as the relevant literature has shown that it has a much smaller impact on technology acceptance than perceived usefulness (e.g., Van Der Heijden, 2004), it was not included in this study.

^2^TAM2 differs from the original TAM in that it includes three additional variables: the subjective norm, voluntariness, and image (c.f., Venkatesh and Davis, 2000).
